# A case of pyelonephritis and bacteremia caused by *Candida glabrata* in a patient on sodium glucose cotransporter 2 inhibitor, successfully treated with micafungin

**DOI:** 10.1186/s40780-025-00475-w

**Published:** 2025-08-05

**Authors:** Shoko Sahara, Teruhisa Kinoshita, Norio Takimoto, Keisuke Oka

**Affiliations:** 1 Department of Pharmacy, Kariya Toyota General Hospital, 5-15, Sumiyoshi- cho, Kariya-city, Aichi Prefecture 448-8505 Japan; 2 Department of Clinical Chemistry and Informatics, Graduate School of Pharmaceutical Sciences, Kumamoto University, 5-1, Oehonmachi, Chuo-ku, Kumamoto-city, Kumamoto Prefecture 862-0973 Japan; 3 Department of Infectious Diseases, Nagoya University Hospital, 65, Tsurumai-cho, Showa-ku, Nagoya-city, Aichi Prefecture 466-8560 Japan

**Keywords:** *Candida glabrata*, Sodium glucose cotransporter 2 inhibitor, Micafungin, Urinary tract infection

## Abstract

**Background:**

Background factors for *Candida* spp. detection in urine include indwelling urinary catheters, diabetes mellitus, and a history of antimicrobial exposure; nevertheless, urinary tract infections caused by *Candida* spp. are usually rare. Fluconazole (FLCZ) is a preferable drug for the treatment of urinary tract infections caused by *Candida* spp.; however, some cases of urinary tract candidiasis resistant to FLCZ have been observed, making the selection of a therapeutic agent difficult. Recently, an increase in fungal genital infections has been reported alongside the increase in the use of sodium glucose cotransporter 2 (SGLT2) inhibitors. Although these medications have not been shown to increase urinary tract infections, concerns persist that they may promote colonization of the genital tract by *Candida* spp. and cause retrograde urinary tract infections, particularly in women. This is a rare case of *Candida glabrata* induced pyelonephritis and bacteremia in a patient receiving SGLT2 inhibitors, successfully treated with micafungin (MCFG).

**Case presentation:**

A patient in her 70s under active treatment for breast cancer was diagnosed with a urinary tract infection and bacteremia caused by *C. glabrata*. The patient was taking SGLT2 inhibitors, and had no history of urinary catheter placement or antimicrobial exposure. In order to avoid the side effects of amphotericin B (AmB) and flucytosine (5-FC), the patient was treated with MCFG and FLCZ for 17 days. No adverse events or recurrence were recorded over the subsequent three months.

**Conclusions:**

Patients taking SGLT2 inhibitors may be more susceptible to urinary tract infections caused by *Candida glabrata*, and in cases of azole-resistant *Candida* spp. urinary tract infection, MCFG may be a treatment option when AmB or 5-FC is difficult to use.

## Background

Most community-onset urinary tract infections are caused by Enterobacterales [1]. Risk factors for the presence of *Candida* spp. in urine include indwelling urinary catheter, diabetes mellitus, and a history of antimicrobial exposure; however, urinary tract infections caused by *Candida* spp. are rare [2]. Sodium glucose cotransporter 2 (SGLT2) inhibitors are effective not only in the treatment of diabetes mellitus but also for heart failure [3] and chronic renal failure [4]. While their use is increasing owing to their broader therapeutic benefits, an increased risk of fungal genital infection has also been reported [5]. Although fluconazole (FLCZ) is typically recommended for urinary tract infections caused by *Candida* spp. because adequate urine concentration of FLCZ can be achieved, azole-resistant *Candida* spp. such as *Candida glabrata* have been occasionally detected in urine [6]. For urinary tract infections caused by *Candida* spp. resistant to azole antifungals, amphotericin B (AmB) and flucytosine (5-FC) are recommended by the Infectious Diseases Society of America (IDSA) [7]. Currently, there is a lack of large-scale data regarding the efficacy and safety of micafungin (MCFG) for management of urinary tract infections caused by *Candida* spp. MCFG is not usually recommended for urinary tract infections due to poor penetration in the tissues of the urinary tract [2, 7]. Further, AmB has side effects such as nephrotoxicity and electrolyte abnormalities, while, 5-FC has a risk of myelosuppression, limiting their use in many patients [7].

Previously, 15 cases of urinary tract infection caused by *C. glabrata* have been reported. Among these, three patients were taking SGLT2 inhibitors, and six were treated with MCFG. However, to the best of our knowledge, no cases have been reported in which patients taking SGLT2 inhibitors were initially treated with MCFG. Here, we report a case of community-acquired urinary tract infection and bacteremia caused by *C. glabrata* in a patient taking an SGLT2 inhibitor, which was successfully treated with MCFG.

## Case presentation

A woman in her 70s (height: 147 cm, weight: 55 kg) had pre-existing left breast cancer, hepatitis B carrier, diabetes mellitus(using an SGLT2 inhibitor and insulin), stage 3b chronic renal failure, and a history of spinal stenosis surgery. There was no history of antimicrobial use in the past three months, and no devices were implanted in her body. The patient was diagnosed with multiple bone and liver metastases 15 years after surgery for left breast cancer and treated with radiation and hormone therapy. Eribulin therapy was initiated owing to the progressive disease. Tenofovir was also initiated to prevent hepatitis B virus reactivation. On day 0 (day 17 of the second course of eribulin), the patient presented to our hospital with fatigue, fever, and lightheadedness. Physical examination findings at the time of admission were as follows: body temperature 38 °C, blood pressure 145/100 mmHg, pulse rate 115/min, respiratory rate 16/min, Glasgow Coma Scale: Eye 4, Verbal 4, Motor 6, and percutaneous arterial blood oxygen saturation 95%. The laboratory results were shown in Table 1.

**Table 1:** Basic laboratory values upon admission

Sodium (mmol/L)	135
Potassium (mmol/L)	4.4
Chlorine (mmol/L)	106
Calcium (mg/dL)	9.9
Magnesium (mg/dL)	2.0
BUN (mg/dL)	16.5
Creatinine (mg/dL)	1.06
eGFR (mL/min/1.73m^2^)	39.3
AST (U/L)	24
ALT (U/L)	19
Albumin (g/dL)	3.5
Glucose (mg/dL)	546
HbA1c (%)	9.6
Ketone (µmol/L)	5701
CRP (mg/dL)	13.48
WBC (x10^3/µL)	2.9
Hemoglobin (g/dL)	12.8
Platelets (x10^3/µL)	267
Neutrophil (/µL)	638

BUN: Blood urea nitrogen

eGFR: estimated glomerular filtration rate

AST: Aspartate aminotransferase

ALT: Alanine aminotransferase

HbA1c: Hemoglobin A1c

CRP: C-reactive protein

WBC: White blood cell count

**Fig. 1:**
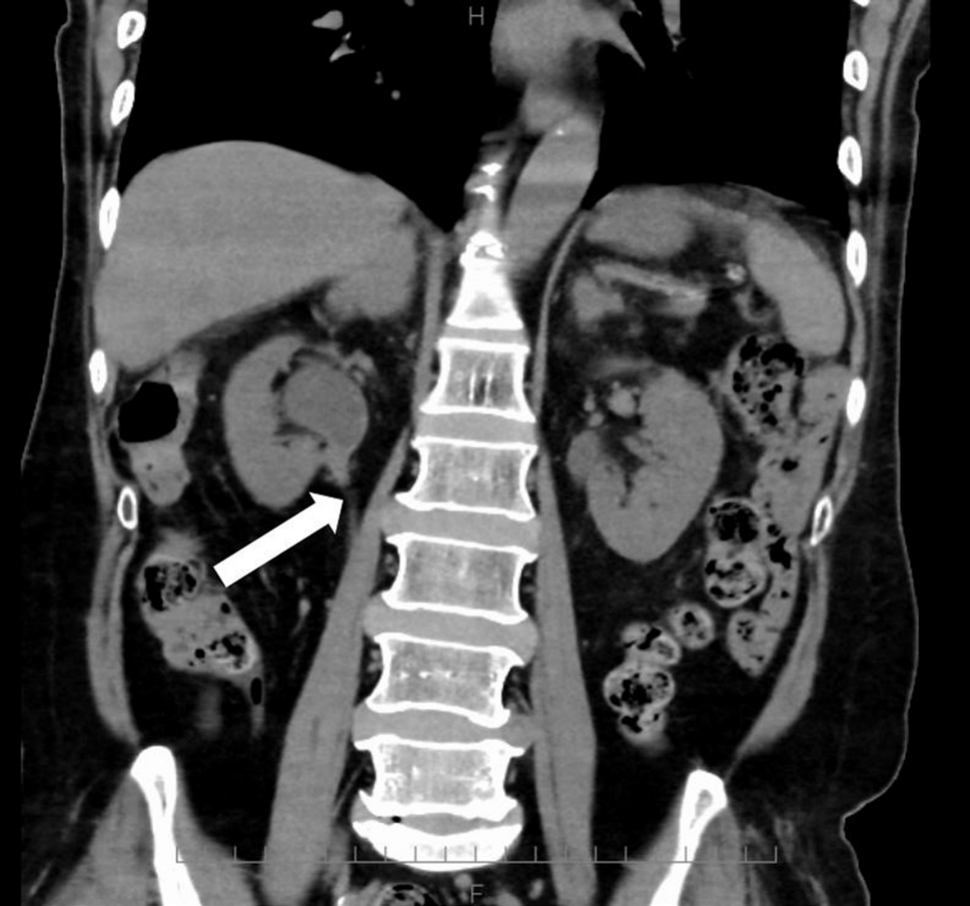
Computed tomography image on day 0. Origin of obstruction in the right ureteral transition and dilatation of the right renal pelvis (arrow)

**Fig. 2:**
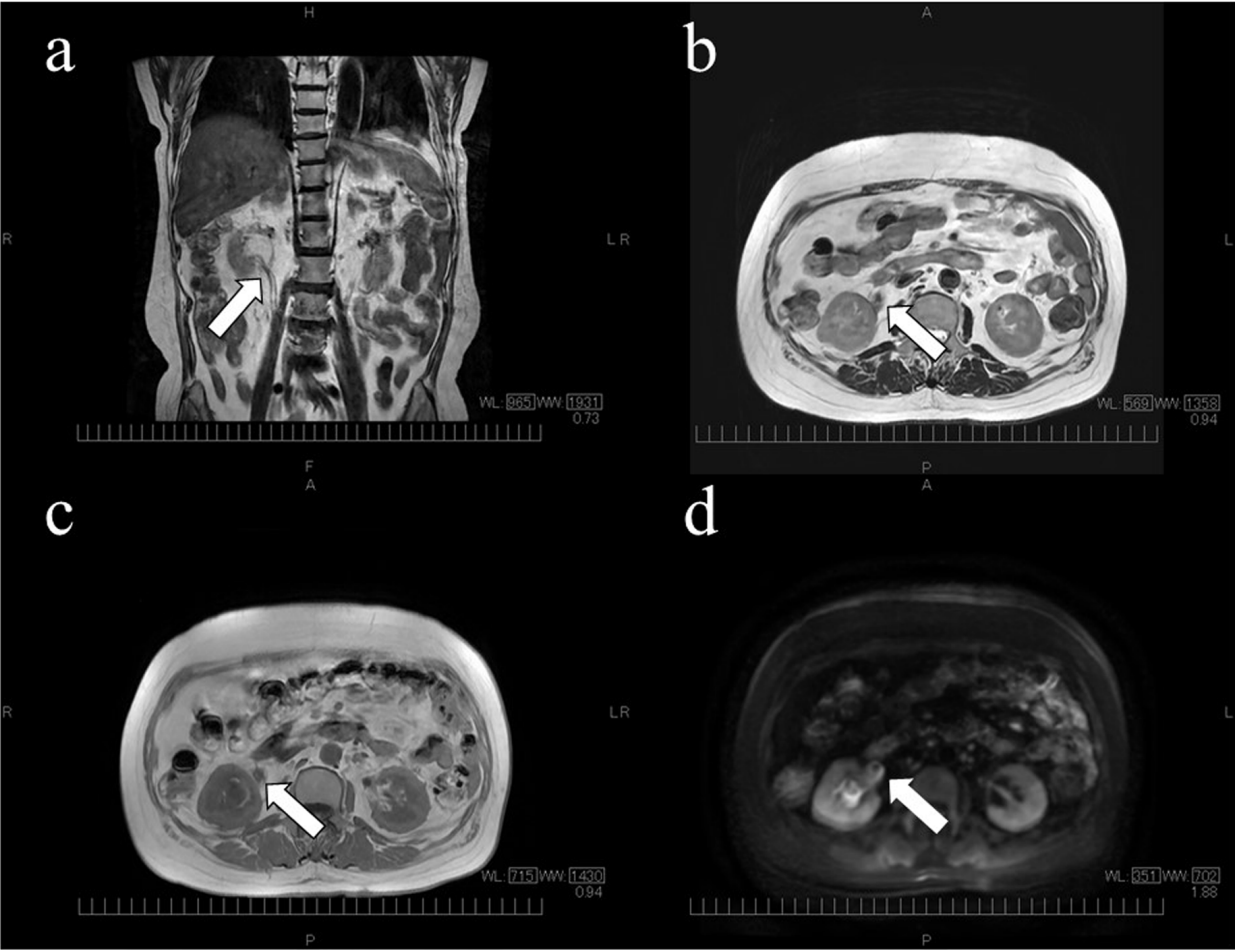
Nuclear magnetic resonance imaging on day 2. (**a**) T2-weighted coronal image, (**b**) T2-weighted axial image, (**c**) T1-weighted axial image, (**d**) Diffusion-weighted image The right renal pelvic ureteral transition area was suspected of inflammatory lesions and debris (arrow)

Urine examination revealed protein 1+, sugar 4+, occult blood 2+, leukocytes 3+, and ketones 3+. The patient had pyuria and pain during urination. Computed tomography showed the origin of obstruction in the right ureteral transition and dilation in the right renal pelvis, with no apparent presence of urinary stones (Fig. 1). The patient was admitted to the hospital with pyelonephritis and hyperglycemic ketoacidosis. Since the infection occurred during chemotherapy administered for cancer, tazobactam/piperacillin (18 g/day) was initiated considering infection with *Pseudomonas aeruginosa* and extended-spectrum β-lactamase-producing *Escherichia coli*. On day 2, the right renal pelvic ureteral transition zone lesion was evaluated using nuclear magnetic resonance, and low-intensity T2 and T1 signals were observed. Diffusion-weighted images were not hyperintense, and no neoplastic lesions were suspected, speculating inflammatory lesions and debris (Fig. 2). Urine cytology results were negative for the tumor. The fever resolved early after admission, and her general condition tended to show improvements. Ureteral stent insertion was not performed because the patient had normal urine output. On day 5, *C. glabrata* was detected in the blood culture (aerobic bottle) collected on the day of admission. The urine culture test confirmed the presence of *C. glabrata* and *Lactobacillus* spp. Treatment with MCFG (150 mg/day) was initiated for pyelonephritis and bacteremia caused by *C. glabrata*. Tazobactam/piperacillin was discontinued on day 6. Transthoracic echocardiography revealed no apparent vegetation, and ophthalmologic examination revealed no intraocular inflammation. On day 7, blood cultures were retested. Owing to a concern that the hydronephrosis would deteriorate in the future along with cancer progression, the right ureteral stent placement was performed. *C. glabrata* was detected in urine collected from the right renal pelvis. The minimum inhibitory concentration (MIC) of each drug against *C. glabrata* is listed in Table 2.

**Table 2:** Drug susceptibility of *Candida glabrata* (based on the clinical and laboratory standards Institute M27 and M44S ED3)

Abbreviation	Drug	Minimal Inhibitory Concentration (µg/mL)
**5-FC**	Flucytosine	≤ 0.125
**FLCZ**	Fluconazole	16
**ITCZ**	Itraconazole	1
**VRCZ**	Voriconazole	0.5
**MCFG**	Micafungin	≤ 0.03
**AmB**	Amphotericin B	1

Blood cultures retested on day 7 were negative. On day 16, the patient was doing well and requested an early discharge. The patient was switched to FLCZ (400 mg/day) and discharged. After a total treatment period of 17 days (14 days after negative confirmation), the patient was cured, and treatment was terminated. Urine culture was retested on day 27 to confirm the absence of *C. glabrata*, and it was not detected. Two months later, the patient visited an ophthalmologist, and no findings of endophthalmitis were reported. No adverse events attributable to MCFG or FLCZ were observed during the treatment period.

## Discussion and conclusions

The use of SGLT2 inhibitors is increasingly recommended by various guidelines owing to their demonstrated efficacy in the treatment of heart failure [3] and chronic renal failure [4], as well as their hypoglycemic effects. At the time of introduction, there was concern about an increased incidence of urinary tract infections due to increased urinary glucose excretion; however, no such association was observed between SGLT2 use and urinary tract infections, according to the findings of a meta-analysis [8]. In contrast, a significant increase in fungal genital infections, predominantly those caused by *Candida albicans* and *C. glabrata*, has been reported in previous studies. Therefore, women and patients with a history of genital infections should be cautioned against genital infections caused by *Candida* spp [5].

A PubMed search conducted on May 24, 2025, using the terms “*Candida glabrata*” and “urinary tract infection” identified a total of 15 case reports of urinary tract infection caused by *C. glabrata* in adults. The majority of reported cases involved female or patients with diabetes; however, only three were reported to be taking SGLT2 inhibitors (Table 3).

**Table 3:** Summary of previous case reports of urinary tract infections caused by *Candida glabrata*

Case No.	Author and year of report	Age	Sex	Blood culture	Under cancer chemotherapy	Diabetes	Therapeutic Agents	Dose per day	SGLT2 inhibitor use	Treatment outcomes
1	Tüz et al.[9]	67	Male	Positive	No	Yes	L-AMB FLCZ	5 mg/kg 400 mg	Unknown	Survived
2	Zhou et al.[10]	85	Female	Unknown	No	Yes	CPFG FLCZ + 5-FC	Unknown Unknown	Unknown	Survived
3	Villegas et al.[11]	46	Female	Unknown	No	Yes	FLCZ MCFG + AmB (topical)	400 mg 100 mg + 50 mg	No	Survived
4	Zeelenberg et al.[12]	Unknown	Male	Unknown	Unknown	Unknown	Unknown	Unknown	Yes	Unknown
5	Deng et al.[13]	21	Female	Negative	No	No	FLCZ FLCZ VRCZ MCFG CPFG	200 mg 300 mg Unknown 50 mg 50 mg	No	Survived
6	Szymankiewicz et al.[14]	66	Male	Positive	No	Unknown	CPFG ANFG CPFG L-AMB	50 mg 100 mg 100 mg 5 mg/kg	Unknown	Died
7	Woloshuk et al.[15]	67	Female	Positive	No	Yes	FLCZ AmB MCFG AmB (topical)	Unknown Unknown Unknown Unknown	Yes	Survived
8	Nagata et al.[16]	52	Male	Positive	No	Yes	MCFG VRCZ	150 mg 300 mg	Unknown	Survived
9	Cases-Corona et al.[17]	53	Female	Positive	No	Yes	CPFG AmB	Unknown Unknown	Yes	Survived
10	Rahimkhani et al.[18]	48	Male	Unknown	No	Unknown	Unknown	Unknown	Unknown	Unknown
11	Balkan et al.[19]	71	Female	Negative	No	Yes	FLCZ	400 mg	No	Unknown
12	Harrabi et al.[20]	64	Female	Negative	Unknown	Yes	AmB	Unknown	No	Died
13	Lagrotteria et al.[21]	39	Female	Unknown	No	Yes	FLCZ MCFG	200 mg 50 mg	No	Survived
14	Lagrotteria et al.[21]	75	Female	Unknown	No	No	MCFG	50 mg	No	Survived
15	Schelenz et al.[22]	71	Female	Positive	No	Yes	L-AMB CPFG AmB (topical)	1 mg/kg 50 mg 1000ug/5mL	No	Survived

AmB: amphotericin B, ANFG: anidulafungin, CPFG: caspofungin, FLCZ: fluconazole, L-AMB: liposomal amphotericin B, MCFG: micafungin, VRCZ: voriconazole, 5-FC: flucytosine

In addition, there were six cases in which MCFG was chosen as the definitive therapy; however none reported the use of SGLT2 inhibitors during initial treatment with MCFG. Of the 15 reports, no cases involved patients undergoing cancer chemotherapy.

Most community-onset urinary tract infections are caused by *Enterobacterales* [1]. Risk factors for *Candida* spp. detection in urine, though rare [2], include indwelling urinary catheters, diabetes mellitus, and a history of antimicrobial exposure. *Candida* spp. is known to develop well in environments with high sugar content. The virulence capacity of *Candida* spp. and its fixation factors are dependent upon host-associated immune factors owing to the complex homeostatic relationship between the fungus and the immune status of the host [23, 24]. A carbohydrate-rich hyperglycemic environment provides essential energy for the formation of biofilms that protect fungal cells from external influences. When urinary glucose is elevated by SGLT2, *Candida* spp. utilizes glucose as a source of nutrients and forms biofilms, which allows it to easily colonize host epithelial cells [24]. The patient had no history of an indwelling urinary catheter or antimicrobial therapy within the three months prior to admission. However, the patient was diabetic, had poor glycemic control, and was taking SGLT2 inhibitors. This may have allowed *Candida* spp. to establish itself in the genitalia and enter the urinary tract in a retrograde manner. The patient was also presented with a narrowing of the urinary tract of an unknown etiology. Considering that *C. glabrata* tends to develop under anaerobic conditions, urinary tract structure may have contributed to pyelonephritis in this case [25]. Eribulin causes neutropenia through microtubule kinetic inhibition, and the time until the neutrophil count reaches a minimum is approximately 13 days after administration [26, 27]. The neutrophil count on admission was 638/µL (day 17 after eribulin administration), suggesting that the patient may have experienced more pronounced neutropenia prior to admission. This neutropenia could have contributed to an increased risk of developing pyelonephritis and bacteremia caused by *Candida* spp.

FLCZ was originally recommended for the treatment of urinary tract infections caused by *Candida* spp [28]. In addition to *C. albicans*,* C. glabrata* and other *Candida* spp. are frequently detected as causative organisms of urinary tract infections [6]. Because *C. glabrata* is often resistant to FLCZ, echinocandin antifungals are recommended as the first-line agents. However, echinocandin antifungals are not recommended for use against urinary tract infections according to the IDSA candidiasis guidelines because of their high protein binding rate and inadequate concentration in the urine [7]. Similarly, liposomal AmB and voriconazole are not recommended because of poor penetration in the tissues of the urinary tract.

Although IDSA guidelines recommend AmB or 5-FC for urinary tract infections caused by *C. glabrata*, AmB is often difficult to use in older adults and patients with renal failure owing to its nephrotoxicity and electrolyte abnormalities. 5-FC exhibits the side effect of bone marrow suppression; safe and effective use of such drugs requires therapeutic drug monitoring, which is not available in Japan. Eventually, in this case, the patient was treated with MCFG, which had fewer adverse events, as AmB was deemed unsuitable owing to renal dysfunction, and 5-FC was avoided to prevent delays in cancer treatment caused by myelosuppression.

A report comparing the outcomes of treatment with echinocandins and FLCZ for urinary tract candidiasis found that echinocandins were not a risk factor for clinical failure [29]. Additionally, a report stated that urinary tract infection caused by *C. glabrata* can be treated with MCFG when urinary Cmax/MIC > 4 [30]. In this report, MCFG was administered at 1.1–3.0 mg/kg/day, and urinary concentrations reached Cmax/MIC > 4 in most cases, indicating successful treatment. Although urinary concentrations were not measured in this case, the administration of 3 mg/kg/day of MCFG was speculated to maintain a relatively high urinary MCFG concentration. MCFG is also known to sufficiently penetrate the renal tissues [31], which may have contributed to its therapeutic effects.

The condition of the patient improved, allowing for a switched to oral FLCZ. The MIC ≤ 32 is considered dose-dependent susceptibility toward FLCZ as per the Clinical Laboratory Standards Institute breakpoint(CLSI) [32]. Once negative blood culture results were obtained, a higher dose of FLCZ was determined to be effective. The area under the concentration-time curve (AUC) of FLCZ in a healthy adult weighing 70 kg is generally considered to be approximately proportional to the daily administered dose. Therefore, administering 400 mg of FLCZ to an adult typically results in an AUC of approximately 400 mg·h/L. According to the report underpinning the CLSI breakpoint determination, achieving an AUC/MIC ratio greater than 25 in the treatment of invasive candidiasis positively correlates with clinical success rates of 91–99% [33].

The patient in this case was diagnosed with stage 3b chronic kidney disease. Patients with impaired renal function have been reported to exhibit approximately half the clearance compared to those with normal renal function [34]. Considering this, the AUC/MIC ratio in this case was estimated to be approximately 50, which explains the observed efficacy of FLCZ in this patient.

Compared to individuals with normal renal function, the blood concentration of FLCZ in this case was estimated to be approximately twice as high. However, FLCZ is generally well tolerated. Although long-term administration of FLCZ has been associated with adverse effects such as alopecia [35], in this case, the treatment was planned for a short duration (only seven days). Therefore, FLCZ was considered to have a favorable safety profile and was chosen accordingly.　Retesting of urine culture on day 27 confirmed negative results.

In conclusion, SGLT2 inhibitors may be a risk factor for urinary tract infections caused by *Candida* spp. In the case of azole-resistant *Candida* spp. urinary tract infection, MCFG may be a treatment option when AmB or 5-FC is difficult to use. However, as this report describes a single case, further accumulation of evidence through case series, retrospective studies, and prospective trials are warranted.

## Data Availability

No datasets were generated or analysed during the current study.

## Abbreviations

IDSA: Infectious Diseases Society of America
AmB: Amphotericin B
FLCZ: Fluconazole
MCFG: Micafungin
MIC: Minimum inhibitory concentration
SGLT2: Sodium glucose cotransporter 2
5-FC: Flucytosine
CLSI: Clinical Laboratory Standards Institute
AUC: Area under the concentration-time curve
